# Editorial: Eating behavior and chronic diseases: research evidence from population studies

**DOI:** 10.3389/fnut.2024.1454339

**Published:** 2024-07-16

**Authors:** Fei Xu, Xiaoyue Xu, Li Zhao, Zumin Shi

**Affiliations:** ^1^Department of Clinical Epidemiology, Jiangsu Province Geriatric Institute, Nanjing, China; ^2^Department of Primary Health Management, Nanjing Municipal Center for Disease Control and Prevention, Nanjing, China; ^3^School of Population Health, The University of New South Wales, Sydney, NSW, Australia; ^4^School of Public Health, Sichuan University, Chengdu, China; ^5^College of Health Sciences, QU Health, Qatar University, Doha, Qatar

**Keywords:** eating behavior, chronic disease, dietary pattern, nutrients intake, nutritional epidemiology, population-based evidence

Non-communicable diseases (NCDs), such as overweight/obesity, cardiovascular diseases (CVDs), cancer, diabetes, and chronic respiratory disease, have been becoming a major global public health problem (1). NCDs account for over 70% of all deaths and impose significant economic burdens worldwide (1). Therefore, it is in urgent need on a global scale to implement effective and feasible actions against NCDs from either public health or economic viewpoint. NCDs are usually preventable, as they share key modifiable lifestyle and behavioral risk factors, including unhealthy eating behavior (1).

As a major lifestyle-related modifiable factor of NCDs, eating behavior is particularly important for the prevention of NCDs. Typically, eating behavior refers to not only dietary patterns but also nutrient intake. From the public health nutrition perspective, population-based evidence on healthy eating is of significance for sharpening policies aimed at preventing NCDs. Thus, this Research Topic was designed to provide population-level evidence on the relationship between eating behavior (both dietary patterns and nutrient intake) and selected NCDs across diverse sub-populations, with particular interest in the interactive associations between eating behavior and other lifestyle/behaviors (e.g., physical activity) in relation to NCDs.

In the paper *Associations of healthy eating index-2015 with osteoporosis and low bone mass density in postmenopausal women: a population-based study from NHANES 2007-2018* (Wang et al.), it was observed that diet quality indicated with healthy eating index-2015 (HEI-2015) was in negative association with the risk of osteoporosis but had no link with low bone mass density (BMD) among postmenopausal women aged 50 years and older in the USA. Osteoporosis, a common metabolic bone disorder, has been emerging as a significant public health issue with a prevalence of 19.7% in the general population worldwide (2). In addition to existing evidence on the association of nutrients intake with osteoporosis and BMD, this study reported the potential link between overall dietary patterns and osteoporosis as well as BMD. It is of important public health meaningfulness to examine the associations of both overall dietary patterns and nutrients intake with osteoporosis.

*The correlation between fruit intake and all-cause mortality in hypertensive patients: a 10-year follow-up study* (Sun et al.). Based data derived from the National Health and Nutrition Examination Survey (NHANES), this cohort study found that, among the common five fruits (apple, banana, pear, pineapple, and grape), intake of apple or banana was associated with decreased risk of all-cause mortality for American hypertensive people. As one of major types of daily foods, fruit is essential to human health. Previously, it has been well-documented that fruit intake was negatively associated with the risk of developing hypertension (3). Meanwhile, it is also important to investigate the relationship between eating behaviors and the risk of death. The present study made a contribution to literature, as it provided another scenario of the association between fruit intake and human health in that increased consumption of specific fruits can reduce the risk of all-cause death for hypertensive individuals.

*Compliance with the EAT-Lancet diet and risk of colorectal cancer: a prospective cohort study in 98,415 American adults* (Ren et al.). With a mean follow-up period of 8.82 years, this study identified that the EAT-Lancet diet (ELD) can reduce the risk of colorectal cancer (CRC) among American adults. ELD, a universally applicable dietary pattern introduced in 2019, encourages the intake of plant-based foods (including vegetables, whole grains, fruits, unsaturated oils, legumes, and nuts) and fish, but limits the consumption of meat and animal products (e.g., beef and lamb, pork, poultry, eggs, and dairy), potatoes and added sugar (4). Different from traditional dietary patterns, the ELD pattern integrated the concepts of nutrition-based health promotion approaches and environmental sustainability (4). In terms of human health promotion, ELD has been examined that it can decrease the incidence and mortality of NCDs such as stroke, CVDs, and cancers (5–8). On the other hand, in terms of environmental sustainability, compliance with the ELD was investigated to be associated with a significant reduction in either greenhouse gas emissions or freshwater consumption (9). Therefore, the ELD may be a scientifically optimized dietary pattern for human long-term development on the earth.

*Soft and energy drinks consumption and associated factors in Saudi adults: a national cross sectional study* (Aljaadi et al.). This study reported a high prevalence of weekly consumption of energy-dense drinks among Saudi adults based on nationally representative data collected in 2021. Energy-dense drinks consumption has been examined to be associated with adverse health outcomes, including obesity, type 2 diabetes (T2D), and CVDs (10–12). It is crucial to implement interventions aimed at reducing the consumption of energy-dense drinks to prevent and alleviate NCDs. The findings regarding energy-dense drinks consumption in this study were similar to those documented in a nationwide survey conducted among Saudi adults in 2013 (13), unfortunately showing that it is not easy for people to modify their preference or habit of food consumption at the population level. Therefore, for the purpose of population-based NCDs prevention through precision lifestyle/behavior intervention, it is important to dynamically investigate population-level eating behaviors and the associated factors.

*Patient-centered nutrition education improved the eating behavior of persons with uncontrolled type 2 diabetes mellitus in North Ethiopia: a quasi-experimental study* (Gebreyesus et al.). This study presented that a 3-month patient-centered nutrition education intervention could significantly improve both specific and overall eating behaviors for T2D patients with HbA1c ≥ 7.0% in Ethiopia. Additionally, the nutritional intervention was effective in lowering the HbA1c levels among the participants in this study. It highlighted that nutrition education as an intervention approach would be effective to improve eating behavior and glycemic control for diabetic patients in a resource-limited country. Adopting and maintaining healthy eating are always encouraged for diabetic patients to effectively manage the blood glucose (14). However, the biggest challenge is not to have people's eating behaviors modified with an intervention program, but to have the favorably-changed eating behaviors maintained for a lifetime or, at least, as long as possible.

Population-based comprehensive lifestyle and behavior intervention is of particular importance and effectiveness for NCDs prevention, and it is often viewed as a feasible and cost-effective approach for preventing NCDs. It is necessary to document the updated findings on the association of lifestyle and behavior with NCDs from nutritional epidemiological studies. The papers related to our Research Topic could offer valuable information to assist researchers, clinicians and policy-makers in designing and implementing dietary-specific intervention programs or policies, thus contributing to the prevention of NCDs.

In summary, eating behavior is time and economic status dependent, which may change as an individual's age or/and socio-economic status changes. This may occur in both developing societies and economically settled communities. Meanwhile, updating the dietary patterns and nutrient intake levels of different sub-populations is also necessary for precision eating behavior intervention. Therefore, although relationships between eating behaviors (dietary pattern, nutrients intake) and specific NCDs have been examined in different societies, studies are always welcome to continuously investigate population-level associations between eating behavior and NCDs in sub-populations with culturally and linguistically diverse background, and especially to further examine the interaction between eating behavior and other factors, such as physical activity, on NCDs. In future, research in these two areas needs to be encouraged to provide evidence supporting healthy dietary guidelines or policies for the prevention of NCDs.

## Author contributions

FX: Conceptualization, Supervision, Writing – original draft, Writing – review & editing. XX: Conceptualization, Writing – original draft, Writing – review & editing. LZ: Conceptualization, Writing – original draft, Writing – review & editing. ZS: Conceptualization, Writing – original draft, Writing – review & editing.
